# Airing Chambers

**Published:** 1866-12

**Authors:** 


### AIRING CHAMBERS.

This may be safely done in winter time when the day is clear, at any hour between sunrise and sunset, but on cloudy and damp days it is better to kindle a fire and thus create a draft up the chimney. A bed should always be made several hours before sundown, before it has had time to gather the damps of the evening.

It will refresh us greatly if on waking up of a winter's night, we get out of bed, throw all the clothing to the foot and the next instant throw it back; this drives all the confined air away from the bedding without allowing it to get very cold; in addition the hands should be passed over the skin of the whole body two or three times; this operation is accompanied with a degree of refreshment and a feeling of purity on entering the bed again which more than pays for the trouble, and it is often a great sleep promoter, enabling a person to fall into a sound slumber in a few minutes after having been tossing restlessly for hours.

Shut your mouth in going from a cold to a hot atmosphere, as well as the reverse; this simple operation brings the temperature of either a cold or hot air to the natural standard before it reaches the lungs, by making it take the circuit of the head; whereas if the mouth is kept open it dashes down upon the lungs like a shock. Whether asleep or awake we should accustom ourselves to keep the mouth shut; the advantage in our sleeping hours is that we do not snore, we don't have the nightmare, flies, bugs and spiders don't crawl down the throat, and we don't tell tales in our dreams; the benefits in the day time are that it induces a more healthful, deep, full and free action of the lungs, prevents innumerable chills and colds, and saves many a domestic sorrow.

